# Injection of ROS-Responsive Hydrogel Loaded with IL-1β-targeted nanobody for ameliorating myocardial infarction

**DOI:** 10.1016/j.bioactmat.2024.12.013

**Published:** 2024-12-24

**Authors:** Lu Wang, Changjiang Yu, Ting You, Xinkui Zhang, Haotao Su, Bihui Cao, Sainiwaer Anwaier, Hongmo Xiang, Chengming Dai, Xiang Long, Linjiang Han, Dengfeng Zhang, Junwei Wang, Peng Zhu, Xinjian Yan, Jialiang Liang, Zerui Chen, Huanlei Huang, Shuoji Zhu, Tucheng Sun, Jimei Chen, Ping Zhu

**Affiliations:** a School of Medicine South China University of Technology, Guangzhou, Guangdong, 510006, China; b Guangdong Cardiovascular Institute, Guangdong Provincial People's Hospital (Guangdong Academy of Medical Sciences), Southern Medical University, Guangzhou, Guangdong, 510100, China; c Guangdong Provincial Key Laboratory of Pathogenesis, Targeted Prevention and Treatment of Heart Disease, Guangzhou Key Laboratory of Cardiac Pathogenesis and Prevention, Guangzhou, Guangdong, 510100, China; d The First Affiliated Hospital, Department of Emergency, Hengyang Medical School, University of South China, China; e Nanfang Hospital, Southern Medical University, Guangzhou, 510515, China; f Guangdong Provincial People’ S Hospital Ganzhou Hospital, Ganzhou, 341000, China

**Keywords:** Myocardial infarction, Responsive hydrogel, IL1β, Nanobody

## Abstract

The cardiac microenvironment profoundly restricts the efficacy of myocardial regeneration tactics for the treatment of myocardial infarction (MI). A prospective approach for MI therapeutics encompasses the combined strategy of scavenging reactive oxygen species (ROS) to alleviate oxidative stress injury and facilitating macrophage polarization towards the regenerative M2 phenotype. In this investigation, we fabricated a ROS-sensitive hydrogel engineered to deliver our previously engineered IL-1β-VHH for myocardial restoration. In mouse and rat models of myocardial infarction, the therapeutic gel was injected into the pericardial cavity, effectively disseminated over the heart surface, forming an in situ epicardial patch. The IL-1β-VHH released from the hydrogel exhibited penetrative potential into the myocardium. Our results imply that this infarct-targeting gel can adhere to the damaged cardiac tissue and augment the quantity of anti-IL-1β antibodies. Moreover, the anti-IL-1β hydrogel safeguards cardiomyocytes from apoptosis by neutralizing IL-1β and inducing M2-type polarization within the myocardial infarction regions, thereby facilitating therapeutic cardiac repair. Our results emphasize the effectiveness of this synergistic comprehensive treatment modality in the management of MI and showcase its considerable potential for promoting recovery in infarcted hearts.

## Highlights

• We synthesis a IL-1β-targeted nanobody(VHH).
• Developed a ROS responsive TSPBA-PVA hydrogel.
• We verified the ability of TSPBA-PVA hydrogel to encapsulate VHH and achieve precise local delivery and selective release.
• Gel-VHHs shown promising therapeutic effects in MI mouse and rat models.

## Graphical abstract

**Figure:**
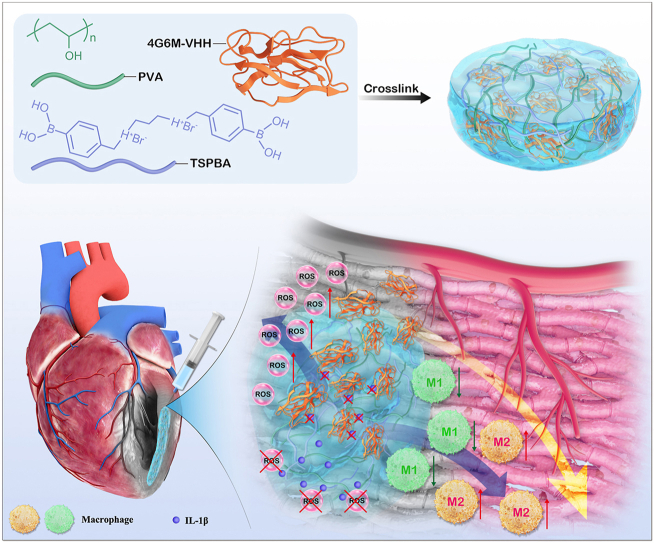

## 1 Introduction

Cardiovascular diseases are recognized as leading causes of mortality globally [1], with myocardial infarction (MI) being a major factor contributing to both mortality and morbidity [2]. Transplantation persists as the sole viable option when heart failure occurs. Notwithstanding recent progressions in mortality metrics, there is an exigency for pioneering therapeutics centered on rejuvenating and rehabilitating impaired cardiac tissues [3]. The crucial role of IL-1β in acute myocardial infarction (AMI) and heart failure (HF) has been verified via preclinical and clinical investigations. Previously, we developed VHH-targeted IL-1β for therapeutic cardiac repair in a mouse MI model.

Nanobodies are therapeutic proteins originated from the heavy chain variable domains (VHH) that naturally exist in the heavy chain-only immunoglobulins of the Camelidae. VHHs possess several advantageous characteristics, including diminutive size, excellent solubility, high thermal stability, accessible epitopes, and robust tissue penetrability [4]. However, the small molecular weight of VHHs results in a reduced half-life, which may compromise their therapeutic efficacy. Intracoronary administration fails to achieve adequate drug accumulation within the injured myocardium due to rapid clearance into the systemic circulation [5]. Therefore, there is an urgent need for a safe and effective strategy for sustained release of VHHs [6].

Injectable hydrogels have gained significant attention for the treatment of MI [7]. These hydrogels show promise in enhancing therapeutic outcomes by facilitating the integration of mechanical and electrical signaling between cardiomyocytes, alongside strategies aimed at mitigating inflammation, promoting cellular proliferation, and addressing fibrotic remodeling following myocardial infarction [8]. Polyvinyl alcohol (PVA) has received approval from the Food and Drug Administration (FDA) for a diverse range of medical applications. Although PVA is not naturally gel-like, it forms a hydrogel when combined with boric acid due to its sensitivity to this compound. N^1^-(4-boronobenzyl)-N^3^-(4-boronophenyl)-N^1^, N^1^, N^3^, N^3^- tetramethylpropane-1, 3-diaminium (TSPBA) is a boric acid-containing cross-linking agent that responds to ROS. Besides rapidly forming a gel with PVA, its key feature is its capacity for automatic degradation in response to ROS stimulation [9,10]. VHH-loaded ROS-responsive hydrogels can effectively scavenge reactive oxygen species, thereby safeguarding cardiomyocytes from oxidative stress. Furthermore, these smart responsive hydrogels facilitate precise, on-demand drug release at the lesion site, enhancing localized therapeutic effects while minimizing administration frequency and associated side effects compared to conventional therapeutic hydrogels [11].

**Fig. 1:**
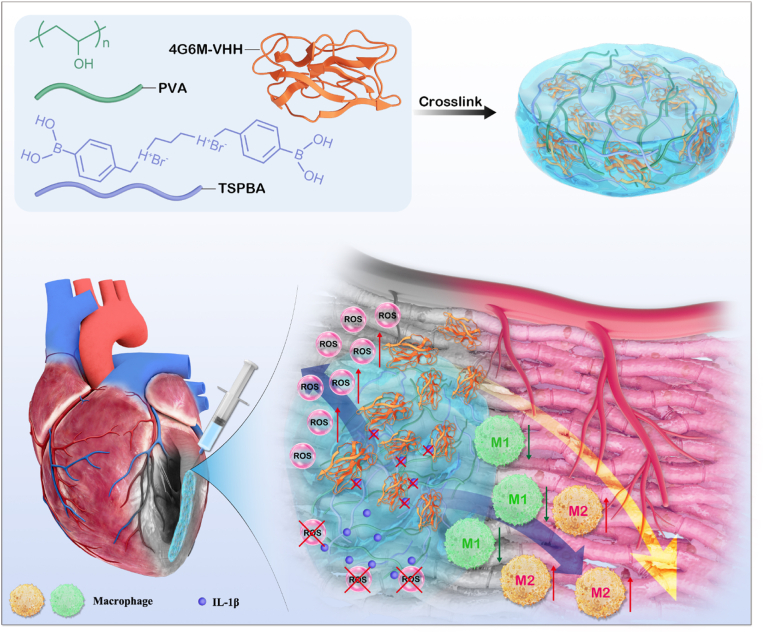
Schematic illustration of the formation and mechanism of the reactive oxygen species (ROS) degradable PVA-TSPBA@VHH hydrogel for eﬃcient myocardial infarction (MI) treatment.

Integrating these design concepts, we developed a ROS-responsive hydrogel loaded with IL-1β-targeted VHH (Gel@VHH), which was subsequently injected directly into the post-infarction pericardial cavity for cardiac repair (Fig. 1). This ROS-sensitive cross-linked polyvinyl alcohol (PVA) hydrogel is engineered to encapsulate 4G6M-VHH and degrades in a ROS-rich environment, facilitating the on-demand release of VHH into the myocardium.

## 2 Materials and methods

### 2.1 VHH construction and purified

The VHH amino acid sequence was meticulously optimized for codon usage and subsequently converted into a nucleotide sequence suitable for bacterial protein expression. The VHH constructs were synthesized as gBlocks gene fragments and sequenced accordingly. Soluble VHH present in the supernatant was purified via Ni-NTA (Cytiva, #29148721) affinity chromatography. The clarified lysate was then applied to a gravity flow column (2 mL bed volume) equilibrated with nickel lysis buffer.

### 2.2 Fabrication of PVA-TSPBA hydrogels

TSPBA (3 wt%) dissolved in ultrapure water was mixed with a 9 wt% aqueous PVA solution at room temperature to produce PVA-TSPBA hydrogels, which were subsequently used in characterization experiments.

### 2.3 Rat and mouse myocardial infraction model

All animal work in this study was compliant with the Guangdong provincial people's hospital. Mice aged 6–8 weeks and rats weighing 160 g were subjected to permanent ligation of the left anterior descending coronary artery (LAD) using 8–0 nylon sutures, approximately 1.0–2.0 mm below the border between the left atrium and left ventricle.

### 2.4 Intrapericardial injection of Gel-VHH and echocardiography in mice and rats

Following left anterior descending (LAD) ligation, PBS, Gel (7.6 mg/kg), and Gel-VHH (0.4 mg/kg VHH) were administered through intracardiac injection. 20 μL or 80 μL of PBS, Gel, or Gel-VHH were injected into the pericardial cavity of mice or rats. Transthoracic echocardiography was conducted using a Philips CX30 ultrasound system equipped with an L15 high-frequency probe by a cardiologist who was blinded to the animal group assignments. All animals inhaled a 1.5 % isoflurane-oxygen anesthesia mixture while in the supine position at 3, 7, 14, and 28 days (n = 5 mice or rats per group). Hearts were imaged in 2D long-axis views at the level of the greatest left ventricular (LV) diameter. Ejection fraction (EF) was calculated based on measurements obtained from views of the infarcted area. Left ventricular ejection fraction (LVEF) was calculated from measurements obtained from views of the infarcted area.

### 2.5 Biodistribution of VHH and VHH-loaded gel in MI mice

VHH was pre-labeled with Cy5.5-NHS ester. Mice were euthanized at 0, 2, and 4 days and then harvested for imaging using the IVIS imaging system (n = 3 mice per group). Additionally, hearts were frozen in OCT compound after imaging. Sections with a thickness of 10 μm were prepared at 100 μm intervals, extending from the root tip to the level of ligation. To investigate whether the released VHH bound to cardiomyocytes, VHH was pre-labeled with Alexa Fluor™ 594 NHS ester (Succinimidyl Ester, Invitrogen™) and then loaded into the ROS-responsive gel. After intrapericardial delivery in MI mice, hearts were harvested on day 3, and 10 μm sections were prepared from the apex to the ligation level at 100 μm intervals for histological analysis.

### 2.6 Immunohistochemistry

Histochemical identification of TUNEL-positive cells in the post-MI heart on day 7 was conducted (n = 5) and other detection was carried out on 4 weeks (n = 5). Heart cryosections were fixed in 4 % paraformaldehyde in PBS for 30 min, followed by permeabilization and blocking with a protein blocking solution (DAKO) that contained 0.1 % saponins for 1 h at room temperature. TUNEL (One Step TUNEL Apoptosis Assay Kit, Beyotime, C1089) staining were carried out according to the manufacturer's instructions (In Situ Cell Death Detection Kit, Fluorescein, and Sigma). For other immunostaining including Ki67(9129, CST), CD31(ab222783, Abcam), CD68(ab31630, Abcam), CD206(ab64693, Abcam) and CD16/32(ab223200, Abcam) the samples were incubated overnight at 4 °C with primary antibodies diluted in the blocking solution. Cardiomyocytes were co-stained by anti-alpha sarcomeric actin antibody. After labeling with fluorescence-tagged secondary antibodies, the slides were mounted using ProLong Gold mounting medium containing DAPI (Thermo Fisher Scientific). Vessel densities were calculated as vessel area divided by total area multiplied by 100 %. Five slides were stained for each group, and five randomly selected fields from each slide (n = 5) were analyzed using NIH ImageJ software.

### 2.7 Statistical analysis

All experiments were performed independently a minimum of three times, and the results are expressed as mean ± standard deviation (SD). For comparisons between two groups, a two-tailed, unpaired Student's t-test was utilized. When comparing more than two groups, one-way ANOVA was applied, followed by a post hoc Bonferroni test. The significance levels indicated by single, double, triple, and quadruple asterisks correspond to p-values of <0.05, <0.01, <0.001, and <0.0001 respectively; p < 0.05 was deemed statistically significant.

## 3 Result

### 3.1 Fabrication of VHH-loaded and ROS-responsive hydrogel

**Fig. 2:**
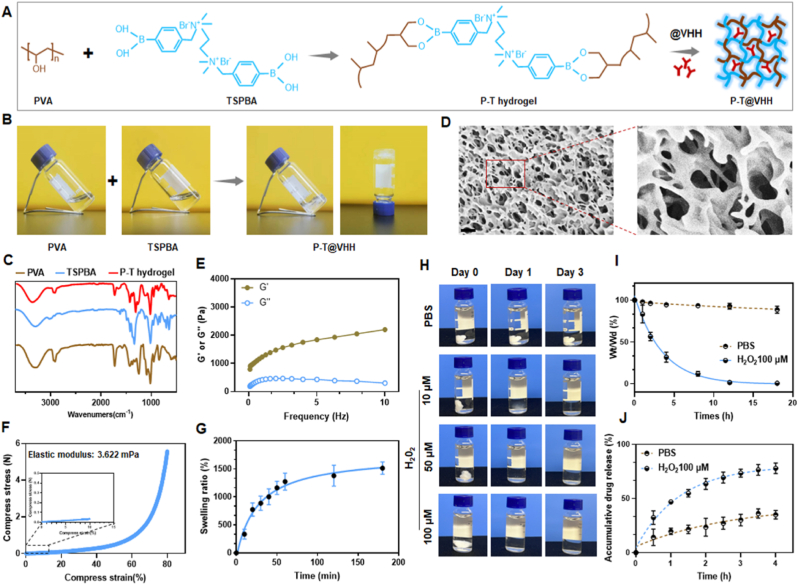
**Characterization of ROS sensitive hydrogels.** (A) The schematic illustrates the composition of the ROS-Responsive Gel@VHH; (B) 9 % (w/v) polyvinyl alcohol (PVA) −3% (w/v) TSPBA complexes shows a rapid sol–gel transition, generating PVA-TSPBA hydrogels; (C) The FTIR characterization of PVA, TSPBA, and P-T hydrogel; (D) The morphology of hydrogel; (E)Rheological frequency sweep of storage (G′) and loss (G″) moduli of the hydrogels; (F) Compression stress strain curves of the hydrogels; (G) Swelling ratio of the hydrogels (n = 3, mean ± SD).(H) ROS-triggered gel disassembly. PVA-TSPBA gel were incubated with ROS at different concentrations; (I) Degradation pattern of the ROS-sensitive hydrogels when immersing in PBS and 100 μM H_2_O_2_ solution (n = 3, mean ± SD); (J)The release profile of VHH from hydrogels under pH 7.4 PBS and H_2_O_2_ solution.

In our previous research, we synthesized three variable heavy chain-only antibodies (VHHs) and validated their affinity for IL-1β, as demonstrated in mouse and rat myocardial infarction (MI) models (Figs. S1 and S2). Given the instability of nanobodies and their rapid degradation during delivery to cardiac tissues, we developed a reactive oxygen species (ROS)-responsive hydrogel (P-T hydrogel). This hydrogel effectively maintains VHH activity within its matrix and releases VHH in response to the elevated ROS levels typical of MI tissues (Fig. 2A). Initially, we synthesized the ROS-responsive linker, N^1^-(4-boronobenzyl)-N^3^-(4-boronophenyl)-N^1^,N^1^,N^3^,N^3^-tetramethylpropane-1,3-diaminium (TSPBA), according to existing literature [12]. and verified its structure using ^1^H NMR (Fig. S4). We then incorporated VHH into the hydrogel during the mixing of polyvinyl alcohol (PVA) and TSPBA solutions, creating the ROS-responsive hydrogel (Gel@VHH) (Fig. 2B and Fig. S3). Fourier-transform infrared spectroscopy (FTIR) confirmed that the hydrogel's spectral peaks encompass those of PVA and TSPBA (Fig. 2C), while rheological testing indicated a predominance of storage moduli (G′) over loss moduli (G″) (Fig. 2E), suggesting a flexible hydrogel formation rather than a viscous liquid. The elasticity modulus of the hydrogel was measured using a universal testing machine. As shown in Fig. 2F, the compressive stress of the hydrogel increased with strain in the 0–80 % range, achieving an elasticity modulus of 3.622 mPa, which falls within the ideal modulus range for injectable hydrogels [13]. Scanning electron microscopy (SEM) observations confirmed the internal porous structure of the P-T hydrogel (Fig. 2D). And the swelling kinetics assays demonstrated a significant swelling ratio, approaching 1500 %, after immersion in PBS for 180 min (Fig. 2G). Given the microporous structure of the hydrogel which facilitates drug loading and release, and the highly hydrated state conferring high biocompatibility, the P-T hydrogel may serve as an ideal platform for the in vivo delivery of nanobodies.

To verify ROS-triggered cleavage of the P-T hydrogel, we immersed the hydrogels in H₂O₂ solutions and monitored changes in their morphology and weight. As illustrated in Fig. 2H and I, disassembly was both concentration- and time-dependent. The gel became almost undetectable after one day in a 10 μM hydrogen peroxide solution and completely dissolved in a 50 μM solution over the same period. At a 100 μM concentration, simulating the MI microenvironment, the hydrogels rapidly lost weight and were undetectable after 18 h. In contrast, immersion in PBS resulted in negligible morphological changes. We then examined the release kinetics of nanobodies. In a simulated ROS-enriched MI microenvironment, the release of VHH was significantly faster compared to PBS, with about 77 % of VHH released within 4 h (Fig. 2J). These findings indicate that the hydrogels undergo ROS-triggered degradation, which likely facilitates on-demand drug release in ROS-enriched MI environments.

### 3.2 Biodistribution of ROS-responsive hydrogel and 4G6M-VHH

**Fig. 3:**
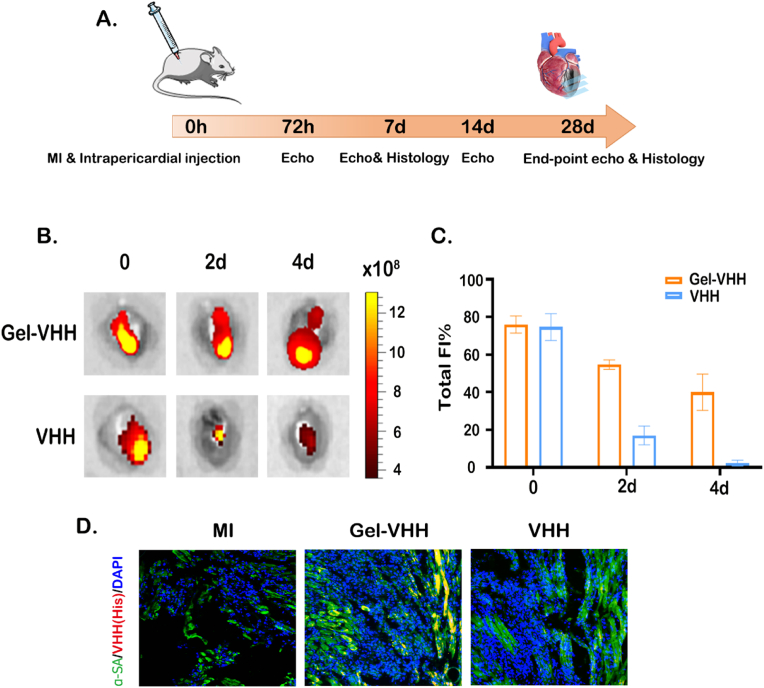
**Intrapericardial injection of Gel-VHH and cardiac retention.** (A) Timeline of animal studies; (B) Ex vivo IVIS imaging of hearts after intrapericardial injection of VHH alone or Gel-VHH at baseline, 2 d and 4 d, n = 3; (C) Quantification of fluorescence intensities of VHH in the hearts; (D) Confocal fluorescence microscopy images showing released VHH into the myocardiμm. Scale bar, 50 μm. White arrows indicate the approximity of released VHH to cardiomyocytes. ∗∗∗∗ indicates p < 0.0001.

Following the in vitro characterization, we initiated the animal study (Fig. 3A). All procedures involving animals were performed in alignment with the protocols established by Guangdong Provincial People's Hospital. Initially, we verified the feasibility of injecting iPC using the hydrogel. During injection, alcian blue was loaded into hydrogels for visualization (Fig. S5). The blue dye spreads across the apex within seconds following the iPC injection of the hydrogel. Subsequently, we evaluated the biodistribution of VHH in MI mice in VHH and Gel-VHH group. The ROS-responsive hydrogel significantly improved the retention of 4G6M-VHH in cardiac tissue compared to 4G6M-VHH administered in saline (Fig. 3B and C). And we proved increased ROS levels after MI (Fig. S6). Furthermore, we examined the fate of injected Gel-VHH through immunostaining of MI heart sections. The released 4G6M-VHH clearly penetrates the epicardium, with some remaining in the pericardial space (Fig. 3D). These findings suggest that iPC injection utilizing ROS-responsive hydrogels is an effective strategy for delivering VHH to injured cardiac tissue and that its biodistribution promotes cardiac repair processes.

### 3.3 Therapeutic efficacy of Gel-VHH in a mouse model of myocardial infarction

**Fig. 4:**
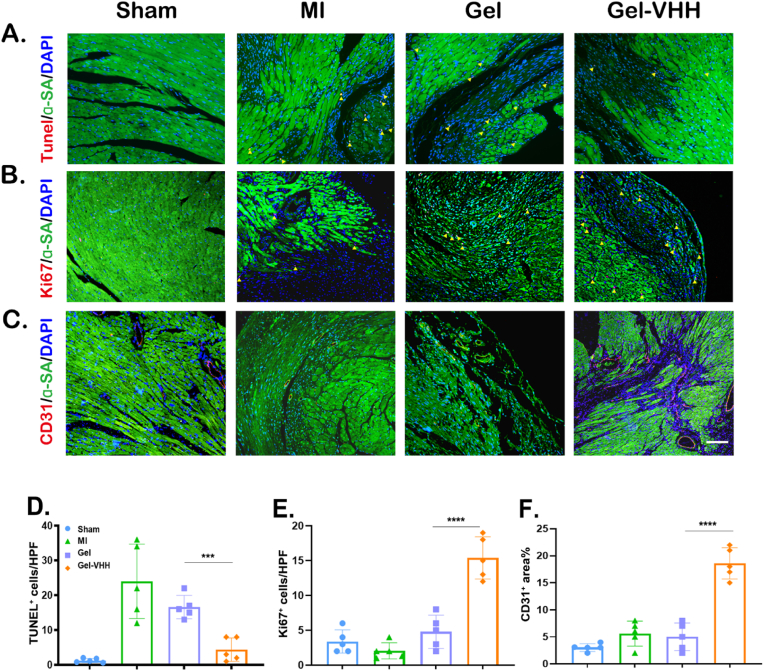
**Gel-VHH injection promotes angiomyogenesis.** Immunostaining of (A)TUNEL on day7 (n = 5), (B) Ki67, and (C) CD31 staining of heart sections 4 weeks after injection. Scale bars, 100 μm. (D–F) Quantitative data corresponding to TUNEL, ki67, CD31 staining (n = 5). ∗∗∗ and ∗∗∗∗ indicates p < 0.005 and p < 0.0001, respectively.

**Fig. 5:**
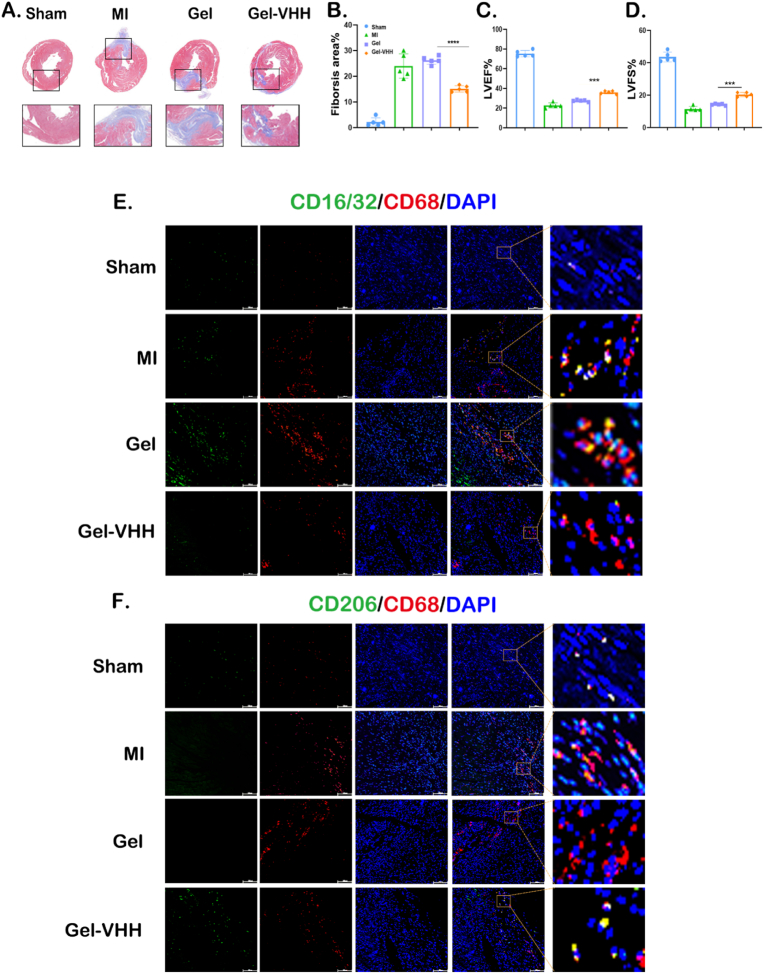
**Functional benefits of Gel- VHH therapy in MI mice.** (A) Representative Masson's stained myocardial sections 4 weeks after treatment; (B) Quantitative analyses of viable myocardiμm from the Masson's images; (C) Left ventricular ejection fractions (LVEFs) and (F) fractional shortening (LVFS) of mouse 4 weeks after treatment (n = 5); (E) Co-immunostaining of pan-macrophage cell surface marker CD68 (red), and the M2 marker CD206 (green) were merged with nuclear staining Hoechst(blue). Scale bars, 100 μm ∗∗ and ∗∗∗∗ indicate p < 0.01 and p < 0.0001, respectively.

We subsequently assessed the therapeutic efficacy of Gel-VHH in mouse cardiac tissue. TUNEL staining revealed a reduction in cardiomyocyte apoptosis within the Gel-VHH group (Fig. 4A–D). Following Gel-VHH treatment, the quantity of Ki67-positive cells in the peri-infarct region was elevated, suggesting that Gel-VHH administration facilitated endogenous cell proliferation. (Fig. 4B–E). Furthermore, Gel-VHH treatment resulted in an increased number of CD31-positive vessels labeled by actin (α-SA) (Fig. 4C–F). Cardiac morphometric analysis of Masson trichrome sections revealed that Gel-VHH administration led to smaller scar areas and a greater extent of viable myocardium, thereby demonstrating its myocardial protective effects (Fig. 5A and B). Echocardiography was conducted at 3, 7, 14, and 28 days post-treatment for MI to evaluate cardiac function following various interventions. In alignment with the observed morphological improvements, Gel-VHH treatment enhanced cardiac morphology and significantly improved left ventricular ejection fraction (LVEF) as well as fractional shortening (FS) (Fig. 5C and D). Additionally, we performed serial semiquantitative analyses of infiltrating macrophages both within the lumen and in the periphery during IL-1β inhibition (Fig. 5E). Moreover, M2 polarization was also observed in the groups treated with Gel-VHH (CD68^+^CD206^+^ at 28 days at peripheral sites) and decreased M1 polarization (CD16^+^/CD32^+^).

### 3.4 Therapeutic efficacy of Gel-VHH in a rat model of myocardial infarction

**Fig. 6:**
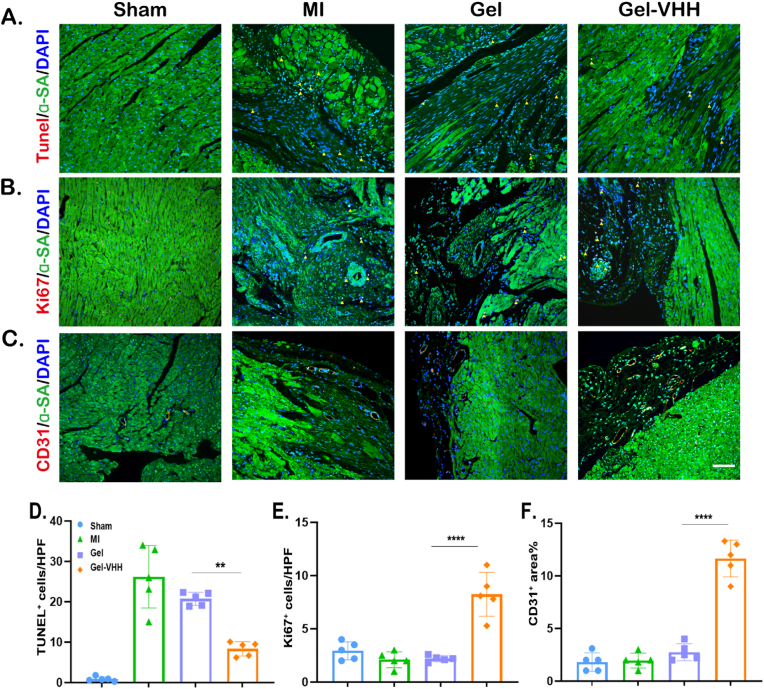
**Gel-VHH injection promotes angiomyogenesis.** Immunostaining of (A)TUNEL on day7(n = 5), (B) Ki67, and (C) CD31 staining of heart sections 4 weeks after injection. Scale bars, 100 μm. (D–F) Quantitative data corresponding to TUNEL, ki67, CD31 staining (n = 5). ∗∗, ∗∗∗ and ∗∗∗∗ indicates p < 0.001, p < 0.005 and p < 0.0001, respectively.

**Fig. 7:**
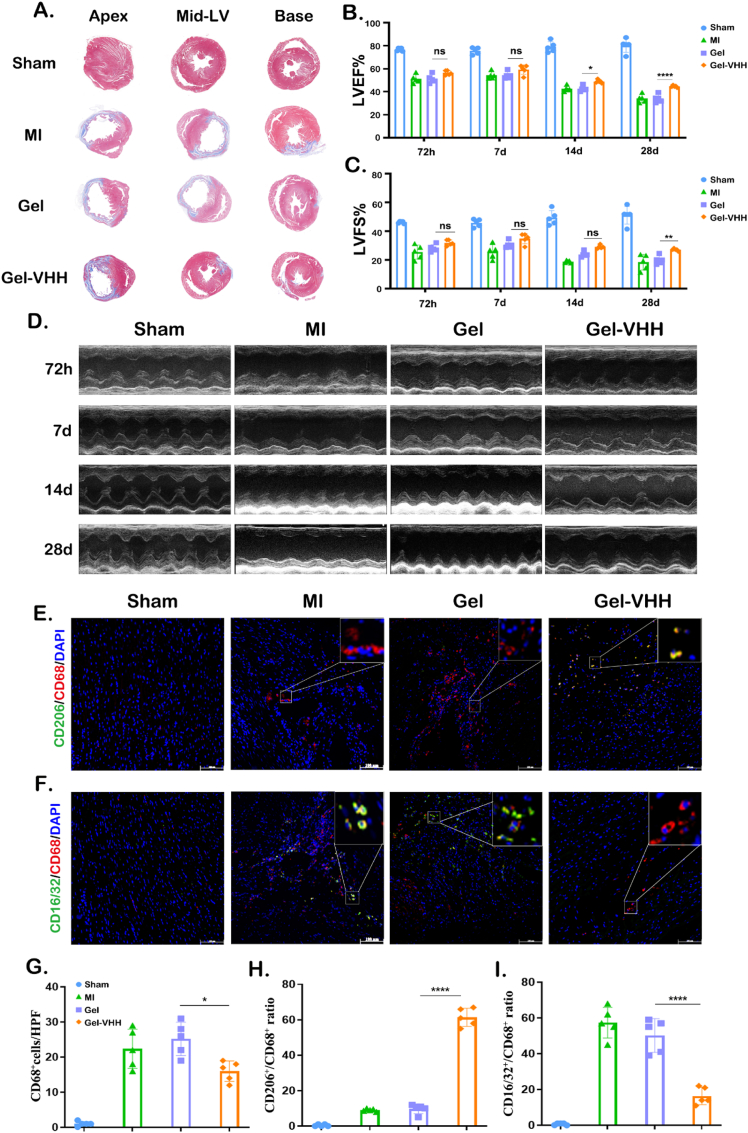
**Functional benefits of Gel- VHH therapy in MI rats.** (A) Representative Masson's trichrome-stained myocardial sections 4 weeks after treatment. (B) Quantitative analyses of viable myocardiμm from the Masson's trichrome images. (C) Left ventricular ejection fractions (LVEFs) and (D) fractional shortening (LVFS) of rat 4 weeks after treatment (n = 5). (E–F) Co-immunostaining of pan-macrophage cell surface marker CD68 (red), and the M2 marker CD206 (green) were merged with nuclear staining Hoechst(blue). Scale bars, 100 μm ∗, ∗∗ and ∗∗∗∗ indicate p > 0.05, p < 0.01 and p < 0.0001, respectively.

Subsequently, we administered Gel-VHH into the pericardial cavity of rats. In alignment with the results in mice, Gel-VHH injection significantly reduced cardiomyocyte apoptosis, as evidenced by TUNEL staining results (Fig. 6A–D). Additionally, Ki67 analysis demonstrated that the administration of Gel-VHH enhanced cellular proliferation (Fig. 6B–E). Simultaneously, Gel-VHH increased the number of CD31-positive vessels, suggesting that it facilitated angiogenesis (Fig. 6C–F). Morphometric analysis of Masson trichrome sections at three cardiac sections demonstrated the protective effect of Gel-VHH treatment, leading to a reduction in scar area. (Fig. 7A). Echocardiographic evaluation of cardiac function at multiple time points post-treatment consistently demonstrated improvements in cardiac morphology, as evidenced by a decrease in left ventricular (LV) hypertrophy (Fig. 7B). Consistent with these morphological benefits, Gel-VHH resulted in increased left ventricular ejection fraction (LVEF) and fractional shortening (FS) (Fig. 7C and D). Furthermore, we conducted a serial semiquantitative analysis of infiltrating macrophages, both within intracavity or periphery of the IL-1β inhibition (Fig. 7E–I). The data indicated that 4G6M-VHH scavenges IL-1β and Gel scavenges reactive oxygen species (ROS) at an earlier stage, these treated groups exhibited a reduced inflammatory response, resulting in a lower CD86^+^/CD206^+^ ratio in Gel-VHH group (Fig. 7E–H). Additionally, Gel-VHH-treated groups exhibited a higher CD16/CD32^+^/CD206^+^ ratio (Fig. 7F–I). Gel-VHH treatment significantly promoted M2 polarization in the rat MI model as well. Therefore, we hypothesized that Gel-VHH application enhances anti-inflammatory effects and promotes cardiac repair.

### 3.5 Regulation of IL-1β transcriptome by TSPBA-PVA gel

**Fig. 8:**
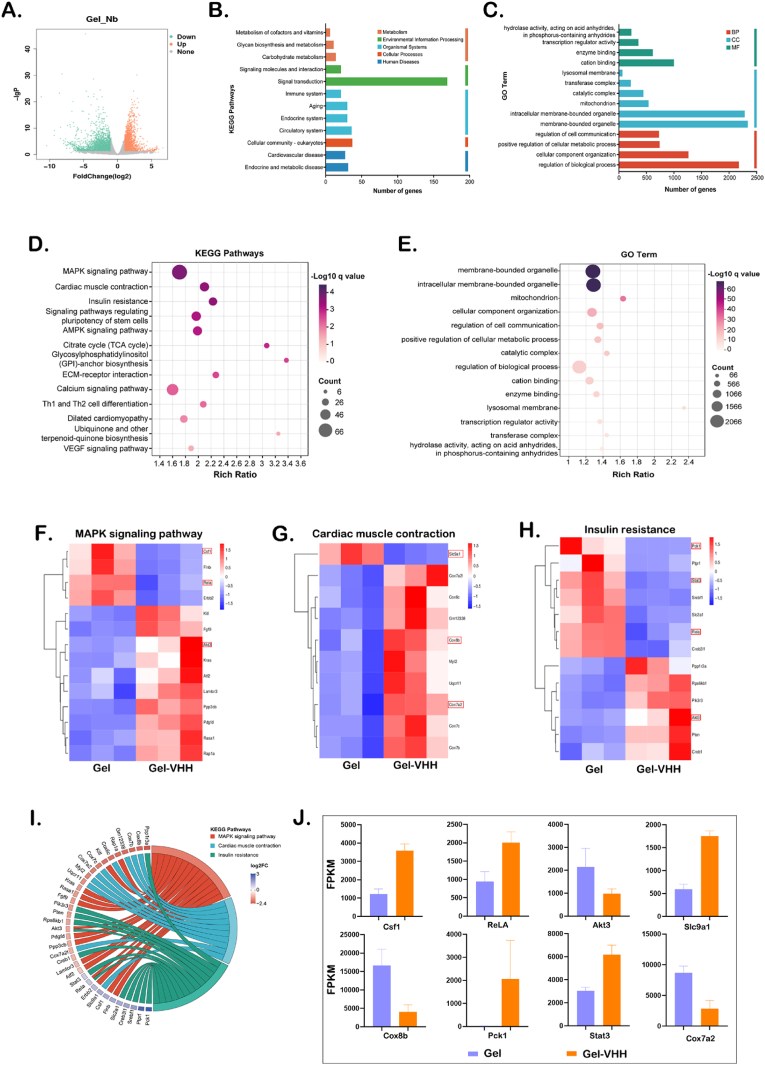
**Transcriptome sequencing analysis of VHH treated with TSPBA-PVA gel.** (A) Volcano plot analyses of total diﬀerentially expressed genes (DEGs) between the Gel treatment group and the Gel-VHH group. Green and orange dots represent down-regulated and up-regulated genes. Grey dots represent the genes not statistically diﬀerent. (B) KEGG analysis of DEGs. (C) GO analysis of DEGs. (D) Bubble plot of KEGG analyses in the “MAPK signaling” pathway. (E) Bubble plot of GO term. Clustering heat map of genes associated with (F)MAPK signaling pathway, (G) Cardiac muscle contraction and (F) Insulin resistance. h) Clustering heat map of genes associated with ABC transporters. (I) KEGG chord diagram in the “MAPK signaling” and “Cardiac muscle contraction” pathway. (J) Fragments Per Kilobase of exon model per Million mapped fragments (FPKM) of some important genes.

Ultimately, we analyzed transcriptome sequencing (RNA-seq) results from the Gel and GEL-VHH groups, to investigate the mechanisms of 4G6M-VHH and TSPBA-PVA gels in myocardial infarction (MI) treatment. In the volcano plot illustrating differences between groups (Fig. 8A), upregulated genes are represented in orange, while downregulated genes are depicted in green. To further elucidate the effects of TSPBA-PVA gel and VHH on the biological functions and gene pathways, as well as to clarify its therapeutic mechanism through neutralization of IL-1β, Gene Ontology (GO) and Kyoto Encyclopedia of Genes and Genomes (KEGG) analyses were conducted (Fig. 8B and C). Functional differences between Gel and Gel-VHH groups primarily centered on ‘metabolic’ pathways, with the ‘environmental information processing pathway’ also playing a significant role in distinguishing these two groups. Subsequently, we conducted KEGG and Gene Ontology (GO) enrichment analyses focusing on the 'Metabolism' pathways, revealing that the most significant differences were observed in the 'MAPK signaling pathway' and 'Cardiac muscle contraction,' as illustrated in Fig. 8D and E. We then generated a chord diagram for KEGG analysis of the 'MAPK signaling pathway' and 'Cardiac muscle contraction' pathways (Fig. 8I). Following this, we performed clustering analysis of MAPK signaling pathway-related genes; the heatmap presented in Fig. 8F demonstrates both upregulated and downregulated genes within these categories. Among these, several genes, including Csf1, Flnb, Atf2, and Lamtor3, were upregulated in the treatment group, while Rap1a, Akt3, Kitl, Kras, and Rasa1 were downregulated. Therefore, we hypothesize that Gel-VHH mitigates IL-1β expression in the injured myocardium and mediates MAPK signaling. Mitogen-activated protein kinases (MAPKs) constitute a class of highly conserved serine/threonine protein kinases that transmit signals via a three-tiered cascade. Up to now, the MAPK signaling pathway has been characterized by four main branches, ERK, c-JNK, p38/MAPK, and ERK5 [14]. These kinases are activated in a sequential manner and collaboratively influence various essential physiological and pathological processes, including the proliferation, growth, and differentiation of cardiac resident cells such as cardiomyocytes, fibroblasts, endothelial cells, and macrophages. Following myocardial infarction (MI), apoptosis of myocardial cells results in reduced cardiac function, whereas the death of non-myocardial cells may worsen scar formation in the heart after MI [14]. Thus, the effective inhibition of apoptosis through the modulation of the MAPK signaling pathway is advantageous. This study indicates that the MAPK pathway may play a pivotal role in restoring cardiac histological integrity and function following treatment with Gel-Nb [15].

Subsequently, we conducted a cluster analysis of genes associated with myocardial contraction, and the heat map (Fig. 8G) revealed that Cox family genes (including Cox7b, Cox8b, Cox7c, and Cox7a2) were significantly downregulated within relevant pathways. Existing literature indicates that these genes may play a role in the progression of heart failure (HF) or other cardiac conditions [[16], [17], [18], [19]]. In addition, the heat map presented in Fig. 8H illustrates genes associated with insulin resistance, reflecting significant alterations in their expression levels. Based on these findings, we constructed a schematic diagram depicting several key genes (Fig. 8I). Insulin resistance is characterized by diminished responsiveness of insulin receptors to insulin, leading to reduced glucose uptake, altered myocardial glucose metabolism, disrupted cardiac electrophysiology, and heightened susceptibility to ischemia-induced myocardial injury [20]. The inflammatory responses observed in acute myocardial infarction (AMI) contribute to the development of insulin resistance, characterized by inflammasome activation that triggers sterile inflammation in response to damaged cells. This process leads to the secretion of IL-1β and the release of locally produced inflammatory cytokines into the bloodstream, ultimately resulting in systemic insulin resistance in subjects treated with Gel-VHH [21]. In the context of Gel-VHH, Pck1 is considered a key mechanism underlying insulin resistance and its modulation of inflammation. Notably, Pck1 was significantly upregulated in subjects treated with Gel-VHH. Additionally, the upregulation of Stat3/RelA interaction contributes to improvements in mitochondrial morphology and function, which may play essential roles in maintaining cellular structure and function in subjects treated with Gel-VHH.

In conclusion, our RNA-seq analysis findings align with previous results. Beyond mediating the MAPK pathway, the treatment group demonstrated the ability to regulate myocardial contraction and insulin resistance, influence the release of inflammatory cytokines, and promote cardiac repair and remodeling.

## 4 Discussion

Myocardial infarction (MI) is one of the leading causes of mortality globally. Enhanced treatment approaches for MI, such as percutaneous coronary intervention, have led to improved survival rates [22,23]. Nevertheless, patients with impaired cardiac function are at a higher risk for developing heart failure (HF), and the increased survival among MI patients further contributes to its prevalence [24]. Heart failure represents a significant portion of cardiovascular-related deaths and is associated with reduced quality of life and considerable healthcare expenses [25]. Consequently, it is essential to create novel treatments focused on preventing heart failure after myocardial infarction by maintaining cardiac function [26,27]. This intense inflammatory reaction is capable of aggravating infarct size and hampering cardiac function [28,29]. Both infarct size and cardiac function constitute important long-term indicators of adverse remodeling and the progression of heart failure [30]. Consequently, immediate therapeutic measures through modulation of the post-myocardial infarction inflammatory response may confer substantial advantages to patients with myocardial infarction [31].

Interleukin (IL)-1β, a key mediator of the inflammatory response following myocardial infarction (MI), has been demonstrated to directly and synergistically compromise cardiac contractility [[32], [33], [34]]. Mechanistic studies suggest that IL-1β signaling and pyroptosis are critical in exacerbating initial ischemic injury, resulting in increased infarct size and reduced cardiac contractility, thereby elevating the risk of heart failure (HF) [35]. Moreover, elevated levels of IL-1β serve as predictors of adverse outcomes following MI in patients. Similarly, researches have demonstrated that inhibition of IL-1β signaling can reduce infarct size and preserve cardiac function [[36], [37], [38]].

Antibodies are increasingly recognized as a potent therapeutic class for autoimmunity, cancer, and infections due to their exceptional specificity, low toxicity, and straightforward pharmacodynamics [39]. Currently available anti-inflammatory antibodies target cytokines, cytokine receptors, and molecules involved in cell-cell interactions [40]. Nanobodies (VHH), which are therapeutic proteins derived from the smallest antigen-binding domains of naturally occurring heavy chain-only antibodies found in camelids, represent a novel approach [41]. These single-domain antibodies are about one-tenth the size of conventional ones and have advantages such as high solubility, remarkable stability, customizable properties, excellent tissue penetration, and better conjugation than traditional antibodies [42,43]. In vivo studies show that monomeric nanobodies penetrate tissues more efficiently and rapidly but are eliminated quickly via renal filtration [44]. The clinical use of VHH therapy for heart disease is partly limited by their short half-life and suboptimal delivery to the heart. As a non-invasive method of drug administration, iPC injection has been utilized for therapeutic delivery in clinical practice [45,46]. Our findings demonstrate that the IL-1β-targeted VHH is released in responsive to H2O2 in vitro. Similarly, we demonstrated that the incorporation of ROS- responsive hydrogel significantly enhances VHH retention within the pericardial cavity, thereby facilitating its penetration into the epicardium, subsequent binding to the myocardium in both mouse and rat models. Enhanced myocardial angiogenesis, cellular proliferation, and improved cardiac function were noted following the administration of Gel-VHH. The Gel-VHH exhibited sustained release capabilities of VHH upon exposure to elevated levels of reactive oxygen species (ROS) resulting from cardiac injury. Considering the synergistic effects between VHH and hydrogels in heart repair, in conjunction with the minimally invasive properties of this approach, our approach represents a significant progress in cardiac biomaterials and drug delivery.

In summary, the findings of this study indicate that 4G6M-VHH effectively neutralizes IL-1β, presenting a potential therapeutic target for myocardial infarction (MI) treatment. The PVA-TSPBA hydrogel developed herein, capable of encapsulating VHH, demonstrates excellent biocompatibility and responsiveness to reactive oxygen species (ROS). This positions it as a promising candidate for drug delivery in MI therapy. Furthermore, the application of PVA-TSPBA for VHH encapsulation represents an innovative experimental strategy that ensures precise local delivery and selective release of VHH. This characteristic aligns well with the practical requirements for clinical applications of VHH. Consequently, the results from this study hold significant promise for clinical translation and may introduce novel treatment strategies for patients with MI.

## CRediT authorship contribution statement

**Lu Wang:** Writing – review & editing, Writing – original draft, Formal analysis, Data curation, Conceptualization. **Changjiang Yu:** Writing – original draft, Formal analysis, Data curation, Conceptualization. **Ting You:** Formal analysis, Data curation, Conceptualization. **Xinkui Zhang:** Formal analysis, Data curation, Conceptualization. **Haotao Su:** Data curation, Conceptualization. **Bihui Cao:** Data curation, Conceptualization. **Sainiwaer Anwaier:** Data curation, Conceptualization. **Hongmo Xiang:** Methodology. **Chengming Dai:** Resources. **Xiang Long:** Validation. **Linjiang Han:** Validation. **Dengfeng Zhang:** Validation. **Junwei Wang:** Data curation. **Peng Zhu:** Validation. **Xinjian Yan:** Writing – review & editing. **Jialiang Liang:** Writing – review & editing. **Zerui Chen:** Writing – review & editing. **Huanlei Huang:** Writing – review & editing, Conceptualization. **Shuoji Zhu:** Writing – original draft, Conceptualization. **Tucheng Sun:** Conceptualization, Writing – original draft, Writing – review & editing. **Jimei Chen:** Writing – review & editing, Conceptualization. **Ping Zhu:** Writing – review & editing, Writing – original draft, Funding acquisition, Conceptualization.

## Ethics approval and consent to participate

Our research involve experimentation on animals. All animal work was compliant with the Guangdong provincial people's hospital (approval number: KY2023-901). And we confirmed authors' compliance with all relevant ethical regulations.

## Declaration of competing interest

The authors declared that the research was conducted in the absence of any commercial or financial relationships that could be construed as a potential conflict of interest.
