# Supplementary material for: Injection of ROS-Responsive Hydrogel Loaded with IL-1β-targeted nanobody for ameliorating myocardial infarction
Source: Bioact Mater. 2024 Dec 24;46:273–84. doi: 10.1016/j.bioactmat.2024.12.013 (PMC11732248; doi:10.1016/j.bioactmat.2024.12.013)
Supplement: Multimedia component 1 [file mmc1.docx]

**Supporting Information**

**Injection of ROS-Responsive Hydrogel Loaded with IL-1β-targeted nanobody for ameliorating myocardial infarction**

**Materials and methods**

**VHH Analytical Methods**

Protein concentrations were determined by nanodrop (Thermo Fisher, nanodrop2000). For evaluating protein production and purification, sodium dodecyl sulfate (SDS)-polyacrylamide gel electrophoresis (SDS-PAGE) was conducted using 12 or 15% (w/v) SDS-gels, followed by Coomassie staining. On- and off-rates of the fluorescein-specific VHH were determined by bio-layer interferometry using the Octet® R8 System (sartorius, Germany). The assay temperature was set to 30 °C biosensors showed no association with the highest applied VHH concentration. Association and dissociation curves were fitted globally according to a 1:1 bimolecular interaction model. Excluded Streptavidin (SA) biosensors were equilibrated with PBS for 300 s. After blocking with assay buffer (PBS supplemented with 1% (w/v) BSA) (baseline, 300 s), association of the VHH (1:1 dilution series 100nM –10 μM in assay buffer) was performed for 300 s, followed by dissociation in assay buffer for 300 s.

**Elisa assay**

Five mice or rats were sacrificed and the blood were harvested after 28 days of treatment and used for IL-1β ELISA(CSB-E08054m and CSB-E08055r, CUSABIO) analysis.

**Synthesis and characterize of TSPBA-PVA gel and VHH loaded Gel**

TSPBA was synthesized from the quaternization reaction between TMPA and 4-(bromomethyl) phenylboronic acid, PVA-TSPBA gel was prepared by mixing PVA and TSPBA. PVA and 4G6M-VHH were mixed first, followed by the addition of TSPBA. All synthesis and characterize methods of TSPBA-PVA gel and VHH loaded Gel according to the literature.^1^

**RNA sequencing and analysis**

Total RNA was isolated using Trizol (Invitrogen) from the MI mice fed a high-fat Western treated with the Gel (n = 3) or with the Gel-VHH (n = 3). The RNA library was prepared according to Illumina RNA Seq library kit instructions with rRNA reduction and the deep sequencing was performed by the Biolinker Technology (Kunming) Co., Ltd. Quality control and quantification of RNA and library were performed using an Agilent 2100 Bioanalyzer and a Kapa Library Quantification Kit (Kapa Biosystems), according to the manufacturer’s protocol. Libraries were sequenced with the illumina novaseq6000 (2 × 150 bp).

We utilized the DESeq2 Bioconductor R package to identify differentially expressed genes at a 5% false discovery rate (FDR) (*P*≤ 0.05) by applying the Benjamini–Hochberg procedure to adjust *P* values. GENCODE/Ensembl gene IDs mapping to known genes were employed, while those mapping to predicted genes were excluded. GSEA incorporated cutoff-free gene expression data. Significantly enriched pathways were identified with a 5% FDR cutoff, and their enrichment significance was quantified using -log10 of *P*_adj_. A functional annotation analysis of the differentially enriched pathway was carried out using PANTHER and Gene Ontology Consortium (http:// geneontology.org/) software.

**References**

1. Gan, Z. *et al.* Stiffness-tuned and ROS-sensitive hydrogel incorporating complement C5a receptor antagonist modulates antibacterial activity of macrophages for periodontitis treatment. *Bioactive Materials* **25**, 347–359 (2023).


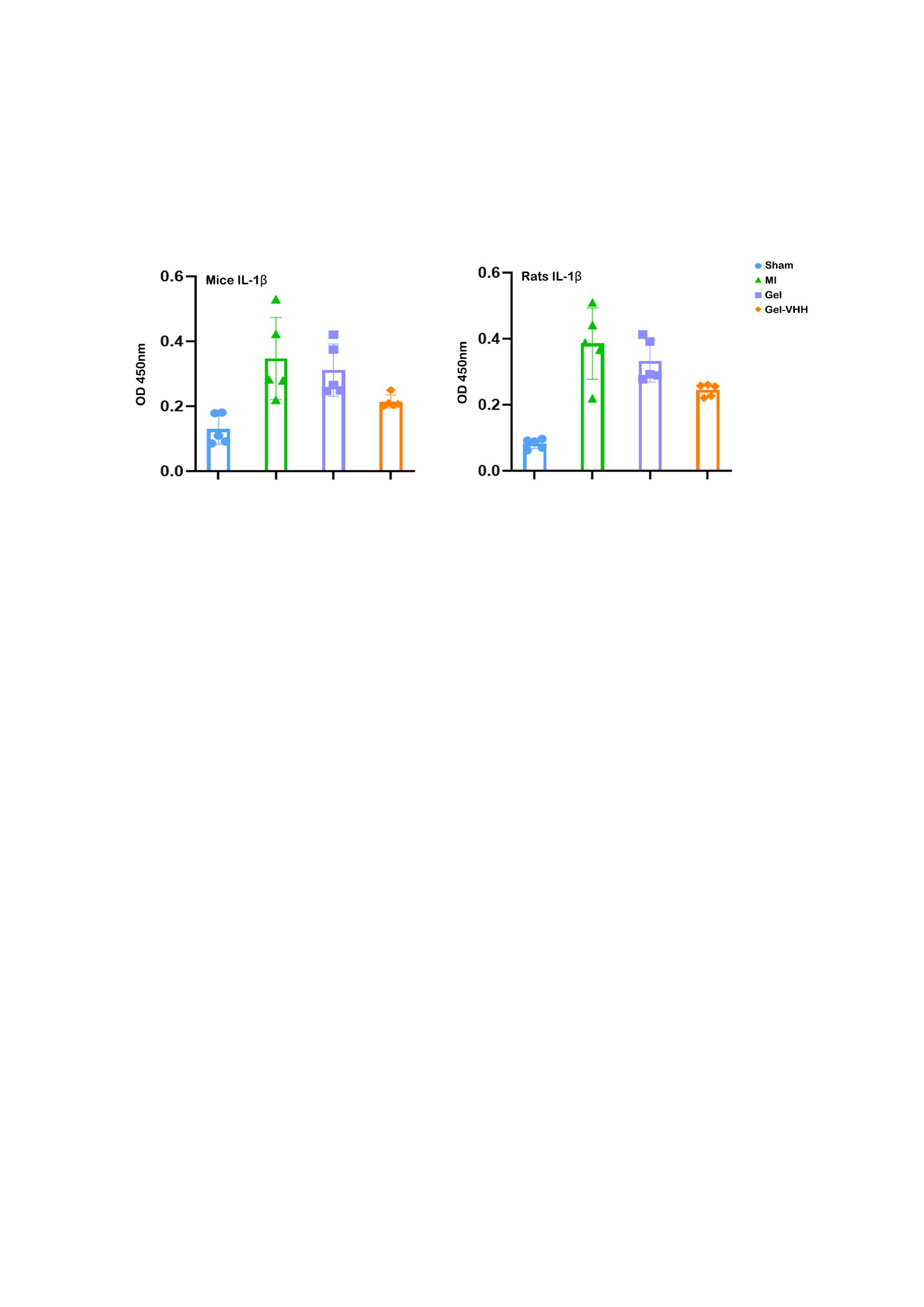


**Figure S1.** **Effects of treatment on IL-1β.** (A-B) Quantitative of the concentrations of mice and rats IL-1β in the blood as detected by ELISA.

**
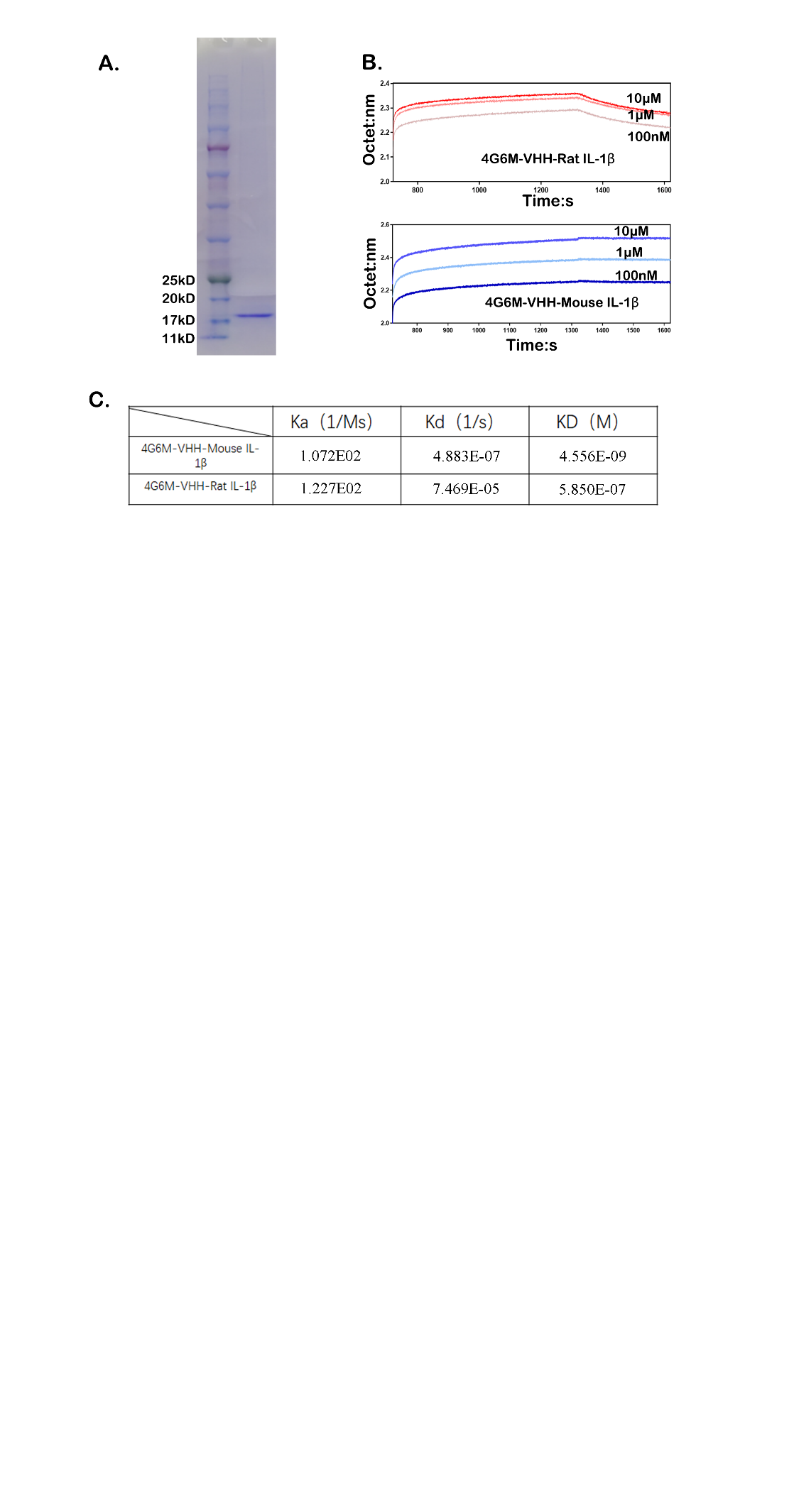
**

**Figure S2. VHH analytical**. (A) SDS-page of VHH. (B)Binding affinity sensograms of VHHs to lL-1β as measured by surface plasmon resonance (BiaCore). (C) VHHs binding affinity table. 'ka’ refers to the association rate constant; 'kd’ refers to the dissociation rate constant, and 'KD’ refers to the affinity constant. Data shown in ELISA represent triplicate repeats.


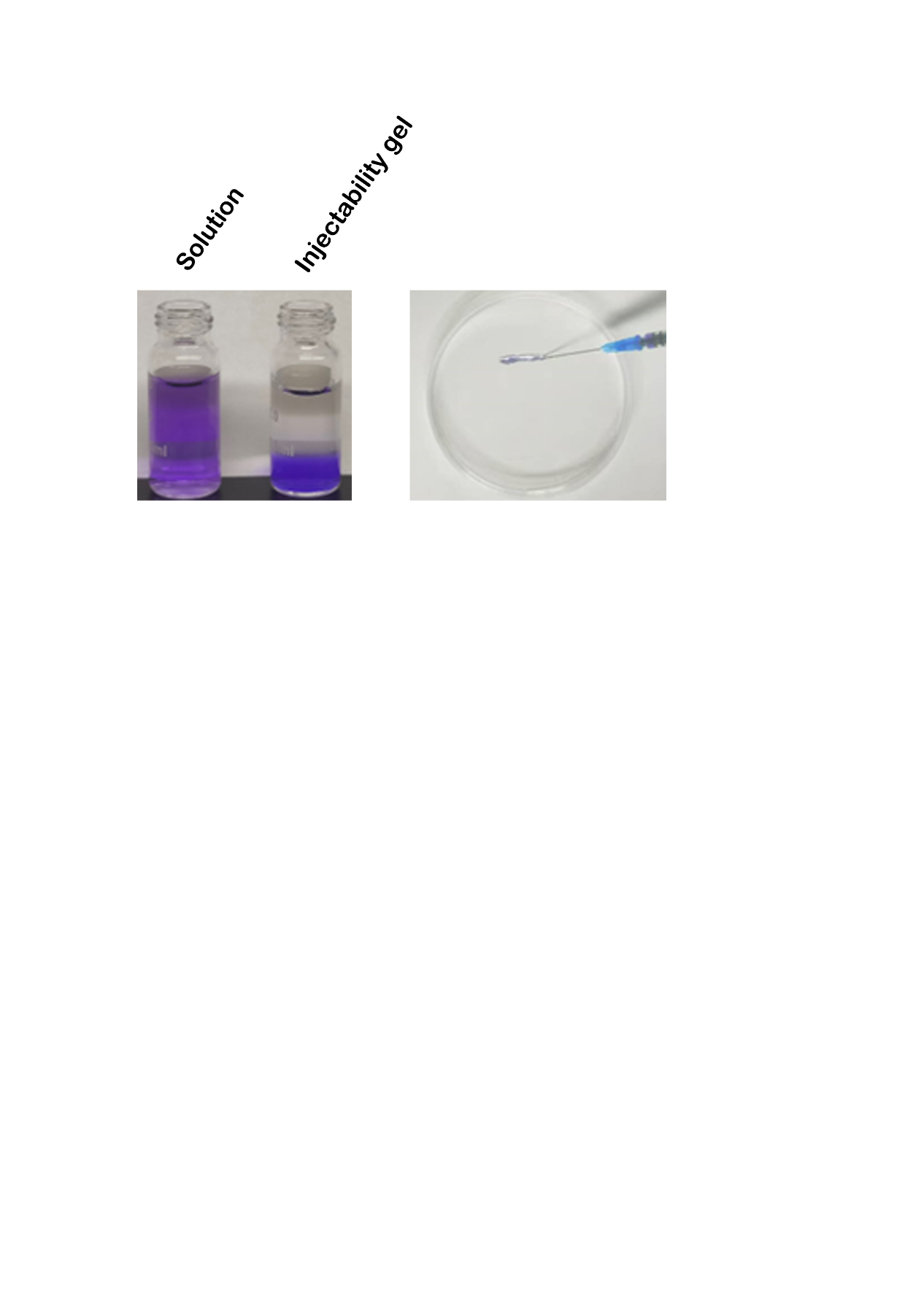


**Figure S3. Photos of the injectability of the hydrogel.**


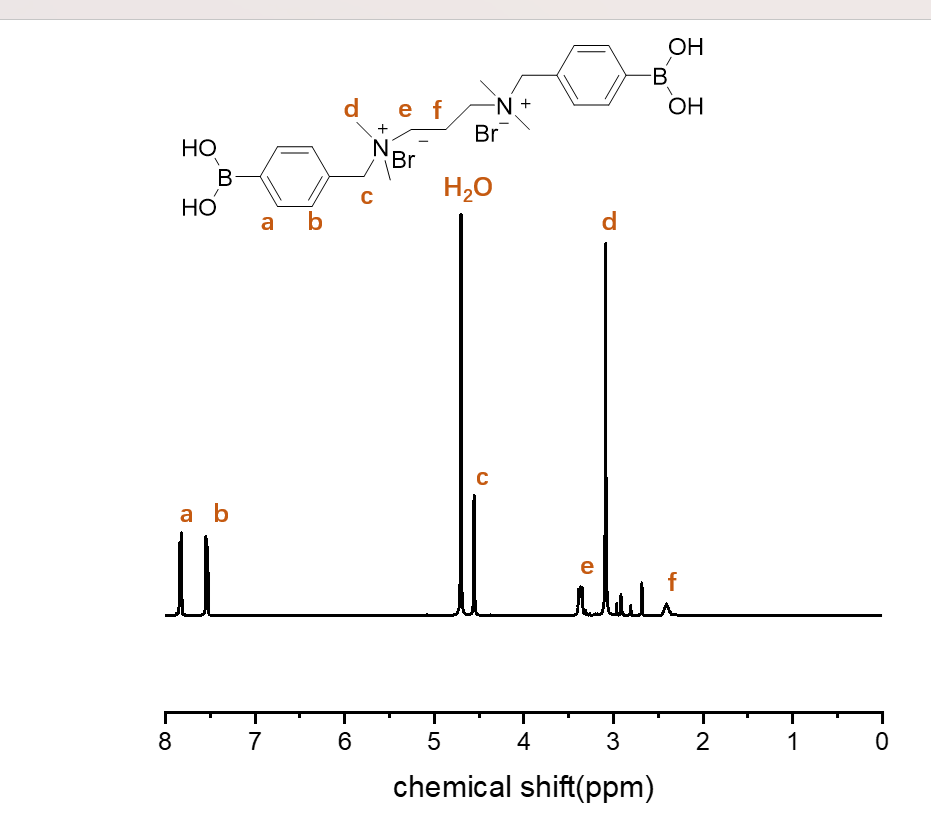


**Figure S4. 1H-NMR of the ROS-responsive linker (TSPBA).**


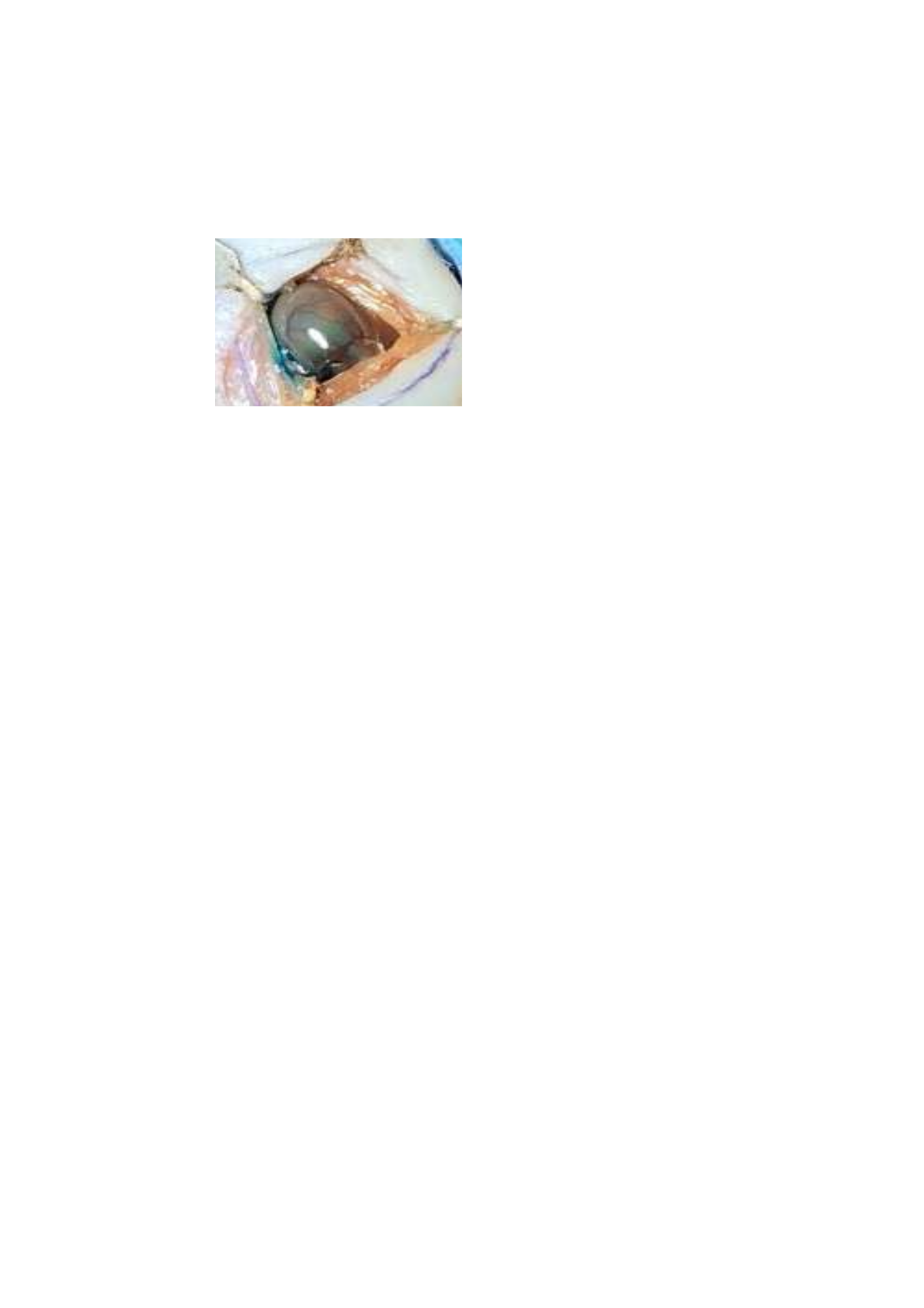


**Figure S5. PVA-TSPBA gel loaded with Alcian Blue on the surface of the heart.**

**
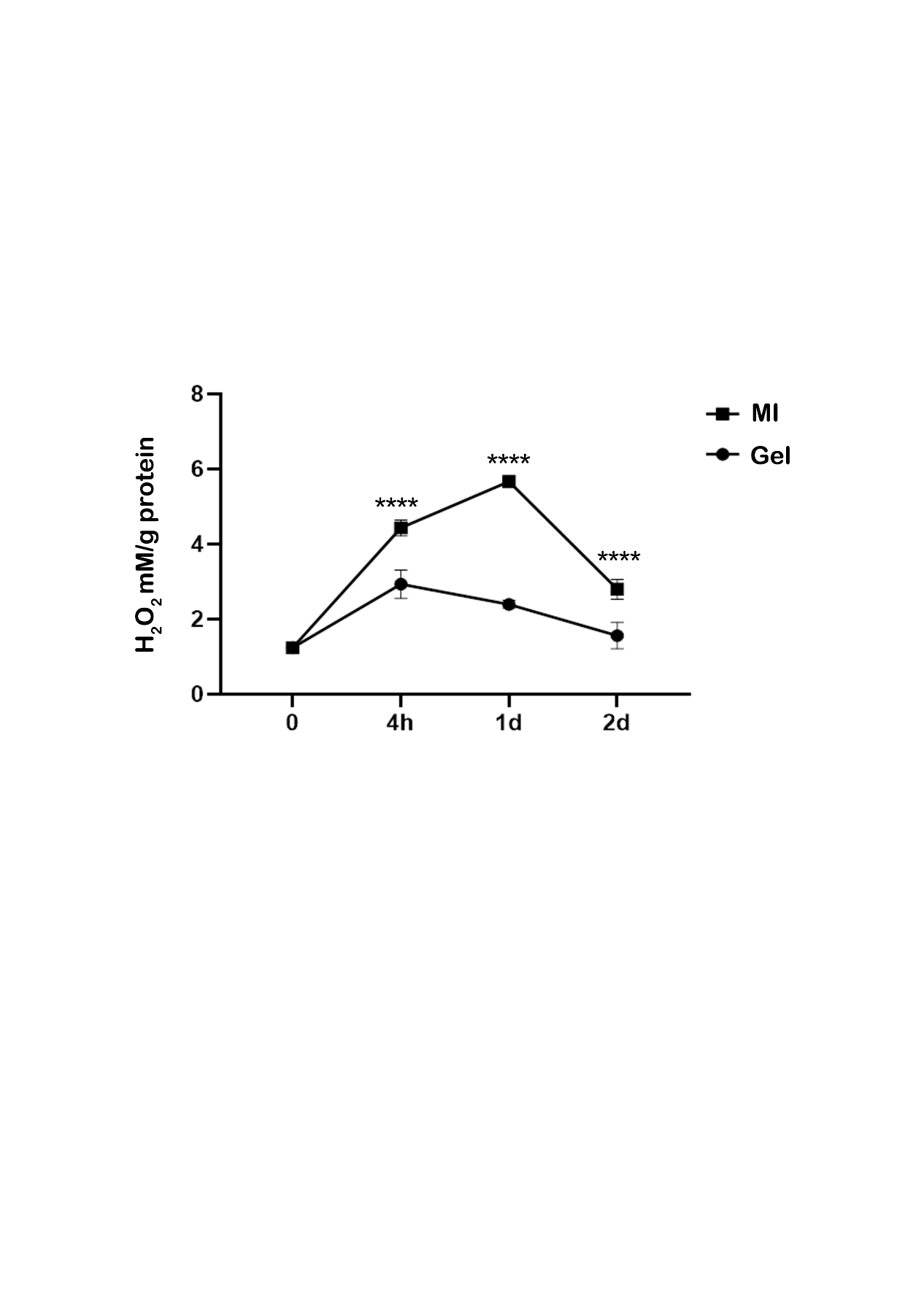
**

**Figure S6. Measurement of H_2_O_2_ concentrations in MI and Gel group heart tissue in Rats model use Hydrogen Peroxide Test Kit(MAK311, Sigma-Aldrich) at different time points.**
